# Can the Open Label Placebo Rationale Be Optimized?

**DOI:** 10.3389/fpain.2021.734882

**Published:** 2021-09-30

**Authors:** Uwe Heiss, Maayan Rosenfield, Michael H. Bernstein

**Affiliations:** ^1^Zeebo Effect, LLC, Burlington, VT, United States; ^2^School of Public Health, Center for Alcohol and Addiction Studies, Brown University, Providence, RI, United States

**Keywords:** placebo, open label placebo, mindfulness, placebo rationale, pain, chronic pain

## Introduction—The Role of the Rationale in Optimizing Placebo Treatment

The success of OLP treatment for chronic pain in clinical trials (1) holds promise for the eventual application of placebo in routine pain management. In preparation for the possibility of a clinical OLP roll-out, it is prudent to optimize OLPs for obtaining the maximum treatment effect. The first-author has previously identified three components (algorithm, rationale, placebo pill) of effective and safe placebo treatment design (2). As shown in Table S1, the algorithm refers to the identification of instances where an OLP may be beneficial and feasible. An algorithm could be implemented by posing a series of questions to the physician or healthcare provider, which would lead to a decision tree that determines if OLPs are suitable. The placebo pill refers to the physical features of the placebo. The focus of this article is the Rationale, which is the explanation given to the patient when administering an OLP.

## State of the Art: Placebo Rationale Practice in Research

The OLP rationale refers to a verbal message wherein patients are told they are receiving a placebo and provided with an explanation regarding why the placebo may work. Almost every study that tested the effect of OLP included a rationale [though see (3)]. As such, patients do not just take a placebo, they are also told why taking a placebo might be efficacious. Both of these elements—the pill and the rationale—are important treatment components (4). In fact, in the only study to date where the presence of a rationale was manipulated, Locher et al. (5) found that OLPs with a rationale reduced experimentally-induced pain more than OLPs without a rationale. However, while this study suggests that including a rationale is important to maximizing the placebo effect, no prior research has examined OLP effects according to different types of rationales. In order to maximize the effect of OLPs, it is important to maximize the impact of the rationale.

In the initial Kaptchuk et al. (6) study, the OLP rationale entailed a 15-min discussion that centered on four points: “(1) the placebo effect is powerful, (2) the body can automatically respond to taking placebo pills like Pavlov's dogs who salivated when they heard a bell, (3) a positive attitude helps but is not necessary, and (4) taking the pills faithfully is critical.” (p. 2). As shown in Table 1, this 4-point discussion has become standard across OLP trials in clinical populations. With few exceptions (7, 8), all studies that examined the efficacy of OLPs outside a dose-extension model have used a rationale almost identical to or a close variation of that used in the Kaptchuk et al. study (9–17). Regarding the exceptions, patients in Kleine-Borgmann et al. (7) simply watched a video describing OLPs and those in Nitzan et al. (8) were told about past efficacy of placebos in studies and that they would likely help alleviate some depressive symptoms.

**Table 1:** Overview of placebo rationales in OLP studies with clinical samples.

**Reference**	***N***	**Condition**	**Standard rationale**	**Rationale components**
Carvalho et al. (9)	83	Chronic low back pain	Yes+	Powerful, conditioning, positive attitude, compliance, video (discussing past efficacy, individual success story)
Hoenemeyer et al. (10)	74	Cancer-related fatigue	Yes	Powerful, conditioning, positive attitude, compliance
Ikemoto et al. (11)	48	Chronic low back pain	Yes+	Powerful, conditioning, positive attitude, compliance, past efficacy
Kaptchuk et al. (6)	80	Irritable bowel syndrome	Yes	Powerful, conditioning, positive attitude, compliance
Kelley et al. (12)	20	Major depressive disorder	Yes	Past efficacy, conditioning, positive attitude, compliance
Kleine-Borgmann et al. (7)	122	Chronic low back pain (independent replication)	No	Video (discussing past efficacy, individual success story)
Kube et al. (13)	54	Allergic rhinitis	Yes+	Powerful, conditioning, positive attitude, compliance, create expectation
Nitzan et al. (8)	38	Unipolar depression	No	Past efficacy, create expectation
Pan et al. (14)	100	Menopausal hot flushes	Yes	Powerful, conditioning, positive attitude, compliance
Schaefer et al. (15)	25	Allergic rhinitis	Yes	Powerful, conditioning, positive attitude, compliance
Schaefer et al. (18)	46	Allergic rhinitis	Yes	Powerful, conditioning, positive attitude, compliance
Zhou et al. (16)	40	Cancer-related fatigue	Yes+	Powerful, conditioning, positive attitude, compliance, past efficacy, create expectation

*Powerful, conditioning, positive attitude, and compliance refer, respectively, to parts 1, 2, 3, and 4 of the standard rationale (see text). “Past efficacy” means there is reference to previous studies that have demonstrated OLP efficacy. “Create expectation” indicates participants were told something similar to “this is likely to help with symptoms of [insert their condition].” Yes+ refers to studies that use Standard rationale with additional component(s). This table excludes studies where the open label placebo is conditioned [e.g., (19–22)], and one study that included an OLP arm but was not designed to study OLP effects (3)*.

## The Possible Moderating Potential of the Rationale

While the Algorithm component of OLP treatment design helps identify which cases or conditions might safely benefit from OLPs, the OLP Rationale and Placebo Pill enable, and possibly modify, the placebo response. The possibility that the OLP response may not just be enabled but moderated by the rationale is broadly consistent with research on deceptive placebos. According to Benedetti (23), “there is not one single placebo effect, but many” (p. 329). Indeed, the placebo effect depends on a variety of factors. For instance, consistent with Table S1 Row 3, placebos that are ostensibly branded are more effective at treating migraine than ostensibly generic placebos (24). Price also influences the placebo effect. In one study, placebos that supposedly cost $2.50 per pill relieved pain in 85% of participants, while placebos allegedly costing $0.10 only relieved pain in 61% of the sample (25). Of particular relevance to the discussion of a rationale, verbal instructions modify the placebo effect. Thomas (17) gave placebos to patients with a minor illness; 2 weeks later, those who were told that they would feel better in a few days improved more than patients who were not given positive expectations. In another study, a negative skin reaction was induced with a histamine skin prick (26). Afterwards, a placebo cream was applied, and those who were told the cream would help had a lower physiological reaction to the allergen than those who were told it would exacerbate the itching. In summary, the effectiveness of deceptive placebos is dependent on situational factors such as verbal instructions. OLP effectiveness may also be moderated by these variables, although no one has yet explicitly examined the role of competing instructions (i.e., rationales).

## An Approach to Rationale Optimization and Individualization

The design of OLP studies thus far is based on rational persuasion conveying a stance that could be described as clinical and authoritative. However, patients' individual dispositions and receptiveness regarding information framing may differ. Some patients may be more receptive to intuitive guidance (i.e., mindfulness) rather than rational persuasion. Patients with an oppositional stance to scientific authority may benefit from being encouraged to suspend disbelief and find out for themselves by observing what happens during their OLP treatment. Therefore, to optimize OLP treatment, we propose two alternative types of rationales: Mindfulness and Suspension of Disbelief. Components of these rationales are provided in Table S2. The potential efficacy of the mindfulness rationale is supported by a meta-analysis of 38 RCTs, where patients assigned to a mindfulness condition reported less pain (SMD = 0.32) compared to those in a control group (typically Treatment as Usual) (27). The potential efficacy of the suspension of disbelief rationale is supported by a pilot study (28) which indicated that while patients are skeptical about the effectiveness of OLPs, they would be willing to suspend disbelief (e.g., “If you say ‘inert pills help you if you take ‘em three times a day.. you'd be like ‘wow, that's weird, but I'll try it… I guess he knows what he's talking about. Can't hurt me.”). Thus, the two new rationales we propose are grounded in the results of earlier research. One consideration of the new aforementioned rationale conditions is that they incorporate guided imagery, which is an effective treatment on its own (29) that may fall under the broad umbrella of mindfulness. The imagery we utilize is OLP-specific; another potential approach would be to dismantle the effect of guided imagery from the proposed rationales. It is likely that each of these components are additive.

## Conclusions and Recommendations

No study so far has examined the efficacy of competing rationales, even though the rationale is an important intervention component and differences in preferences for placebo information have been noted (30). While the rationale developed by Kaptchuk et al., and used widely by others, has been effectively applied, it is possible that patients may respond more positively to other types of rationales. The natural next step in this line of research is to examine the impact of OLP across multiple rationales. Given the large body of work showing that OLPs are effective for chronic low back pain (7, 9, 11) or other chronic pain conditions (3, 6), we suggest this is the appropriate clinical condition to examine rationale efficacy. We propose two additional rationales based on the concepts of mindfulness and suspension of disbelief to evaluate and optimize OLP treatment for chronic pain. Future studies with a clinical population could compare these rationales against each other, as well as to a condition where participants receive an OLP but without a rationale [as done by Locher et al. (5) with healthy volunteers]. This latter design would enable us to distill the effect of the Placebo Pill from the Rationale component [also see (4)]. We also suggest that patient's receptiveness to different rationales may vary with personality traits and patient preferences, marking the beginning of personalized OLP treatment.

## Author Contributions

UH and MB conceived the paper, wrote initial draft, and conceived of the mindfulness and suspension of disbelief rationales. MR created table and edited paper. All authors contributed to the article and approved the submitted version.

## Funding

MB was funded by the National Institutes of Health K01DA048087.

## Conflict of Interest

UH was the CEO and founder of Zeebo Effect, LLC. The remaining authors declare that the research was conducted in the absence of any commercial or financial relationships that could be construed as a potential conflict of interest.

## Publisher's Note

All claims expressed in this article are solely those of the authors and do not necessarily represent those of their affiliated organizations, or those of the publisher, the editors and the reviewers. Any product that may be evaluated in this article, or claim that may be made by its manufacturer, is not guaranteed or endorsed by the publisher.
