# Supplementary material for: Can the Open Label Placebo Rationale Be Optimized?
Source: Front Pain Res (Lausanne). 2021 Sep 30;2:734882. doi: 10.3389/fpain.2021.734882 (PMC8915569; doi:10.3389/fpain.2021.734882)
Supplement: Supplementary file 1 [file Data_Sheet_1.docx]

**Supplementary Material**

| **Table S1.** *Components of Open Label Placebo Treatment Design* | | |
| --- | --- | --- |
|  | **Design Elements** | **Conceptual Basis** |
| **Algorithm** | 1. Is a placebo response physiologically possible? 2. Is the intended use safe? 3. Is OLP in this case ethical? | The placebo algorithm guides the selection of beneficial, safe, and ethical use cases. |
| **Rationale** | 1. General enabler (create awareness, set expectation) 2. Specific to clinical condition (e.g., chronic pain) 3. Matching patient traits & preferences (hypothetical) | The rationale enables the placebo response. Assumption: the rationale modifies treatment effect and can be optimized. |
| **Placebo pill** | 1. Form (e.g., pill, capsule) 2. Features (e.g., color, size, packaging) 3. Perceived value (e.g., brand, price, quality) | Form, features and perceived value of the placebo contribute to the overall treatment effect. |

*Note:* The design of open label placebo treatment comprises three components: Algorithm, Rationale, and Placebo. The elements of each of these are described above.

| **Table S2.** *Rationale Summary* | | |
| --- | --- | --- |
|  | **Primary Components** | **Conceptual Basis** |
| **Standard** | 1. The placebo effect is powerful 2. Placebos can work as a result of conditioning 3. A positive attitude is not necessary 4. Compliance is critical | Several prior OLP RCTs, originating with Kaptchuk et al. (2010). |
| **Mindfulness** | 1. Accept and observe your pain 2. Imagine the placebo pill dissolving your pain 3. Placebos can unlock your body’s natural healing power | Research suggests that mindfulness is efficacious in reducing chronic pain (Hilton et al., 2016). Patients believe in mind-body connection (Bernstein et al., 2021) |
| **Suspension of Disbelief** | 1. 1 in 3 people respond to taking a placebo 2. Keep an open mind about whether the placebo might work for you 3. Create a story about your pain and pain relief | In prior qualitative research, many people are open to trying OLPs but have some doubt about effectiveness (Bernstein et al., 2020; 2021) |

**References (Supplement Only)**

Bernstein, M. H., Fuchs, N., Rosenfield, M., Weiss, A.-P., Blease, C., Locher, C., . . . Beaudoin, F. (2021). Treating pain with open-label placebos: A qualitative study with post-surgical pain patients. *The Journal of Pain*.

Bernstein, M. H., Locher, C., Stewart-Ferrer, S., Buergler, S., DesRoches, C. M., Dossett, M. L., . . . Blease, C. R. (2020). Primary care providers' use of and attitudes towards placebos: An exploratory focus group study with US physicians. *British Journal of Health Psychology*. doi:10.1111/bjhp.12429

Kaptchuk, T. J., Friedlander, E., Kelley, J. M., Sanchez, M. N., Kokkotou, E., Singer, J. P., . . . Lembo, A. J. (2010). Placebos without deception: a randomized controlled trial in irritable bowel syndrome. *PloS one, 5*(12), e15591.
